# Consensus on acromegaly complications: an update

**DOI:** 10.1007/s11102-026-01639-z

**Published:** 2026-04-29

**Authors:** Andrea Giustina, Luigi di Filippo, Maria Fleseriu, Rosario Pivonello, Stephan Petersenn, John Wass, Susan L. Samson, Alberto M. Pereira, Raúl M. Luque, Betina Biagetti, Maria Chiara Zatelli, Ken K Y Ho, Cesar L. Boguszewski, Aart Jan van der Lely, Mark Gurnell, Nienke Biermasz, Katharina Schilbach, Diego Ferone, Monica R. Gadelha, Adriana G. Ioachimescu, Ezio Ghigo, Christian J. Strasburger, Pinar Kadioglu, Pietro Maffei, Niki Karavitaki, Mónica Marazuela, Michael Buchfelder, Sabrina Chiloiro, Anton Luger, Yona Greenman, Elena Valassi, Ignacio Bernabeu, Stefano Frara, Philippe Chanson, Thierry Brue, John Ayuk, Felipe F Casanueva, Annamaria Colao, Pietro Mortini, Sebastian Neggers, Manel Puig-Domingo, Meliha Melin Uygur, Shlomo Melmed

**Affiliations:** 1 Institute of Endocrine and Metabolic Sciences, San Raffaele Vita-Salute University and IRCCS San Raffaele Hospital, Milan, Italy; 2 Pituitary Center, Medicine and Neurological Surgery, Oregon Health & Science University, Portland, OR USA; 3 Università Federico II di Napoli, Naples, Italy; 4 ENDOC Center for Endocrine Tumors, Hamburg, Germany; 5 Oxford Centre for Diabetes, Endocrinology and Metabolism, Oxford University Hospitals, Oxford, UK; 6 Department of Medicine, Division of Endocrinology, Diabetes and Metabolism, Mayo Clinic, Jacksonville, FL USA; 7 Amsterdam UMC, Department of Endocrinology and Metabolism, University of Amsterdam, Pituitary Center Amsterdam, Amsterdam, Netherlands; 8 Department of Cell Biology, Physiology, and Immunology, Centro de Investigación Biomédica en Red de Fisiopatología de la Obesidad y Nutrición (CIBERobn), Maimonides Institute for Biomedical Research of Córdoba (IMIBIC), University of Cordoba, Cordoba, Spain; 9 Endocrinology & Nutrition Department, Hospital Universitario Vall de Hebrón, Barcelona, Spain; 10 Department of Medical Sciences, Section of Endocrinology, Geriatrics & Internal Medicine, University of Ferrara, Ferrara, Italy; 11 Garvan Institute of Medical Research, St. Vincent’s Hospital and the UNSW Sydney, Sydney, Australia; 12 Department of Internal Medicine, Endocrine Division (SEMPR), University Hospital, Federal University of Parana, Curitiba, Brazil; 13 Pituitary Center Rotterdam, Erasmus University Medical Center, Rotterdam, Netherlands; 14 Metabolic Research Laboratories, Institute of Metabolic Science, University of Cambridge, Addenbrooke’s Hospital, Cambridge, UK; 15 Division of Endocrinology, Department of Medicine, Center for Endocrine Tumors Leiden, Leiden University Medical Center, Leiden, The Netherlands; 16 Medizinische Klinik und Poliklinik IV, LMU Klinikum, LMU Munich, Germany & Deggendorf Institute of Technology, Deggendorf, Germany; 17 Endocrinology Unit, IRCCS Ospedale Policlinico San Martino, Università di Genova, Genova, Italy; 18 Neuroendocrinology Research Center/Endocrinology Division, Universidade Federal do Rio de Janeiro, Rio de Janeiro, Brazil; 19 Department of Medicine, Division of Endocrinology and Molecular Medicine, Medical College of Wisconsin, Milwaukee, WI USA; 20 Department of Medical Sciences, University of Turin, Turin, Italy; 21 Department of Endocrinology and Metabolism, Charité- Universitätsmedizin Berlin, Berlin, Germany; 22 Division of Endocrinology-Metabolism and Diabetes, Istanbul University - Cerrahpasa, Istanbul, Türkiye Turkey; 23 Department of Medicine, Padua University, Padua, Italy; 24 Department of Metabolism and Systems Science, College of Medicine and Health, University of Birmingham, Birmingham, UK; 25 Department of Endocrinology and Nutrition, Hospital Universitario de La Princesa, Universidad Autónoma de Madrid, Madrid, Spain; 26 Department of Neurosurgery, University Hospital Erlangen, 91054 Erlangen, Germany; 27 UOC Endocrinology and Diabetology, Fondazione Policlinico Universitario A. Gemelli IRCCS, Roma, Italy; 28 Clinical Division of Endocrinology and Metabolism, Department of Medicine III, Medical University of Vienna, Vienna, Austria; 29 Institute of Endocrinology, Diabetes, Metabolism and Hypertension, Tel Aviv-Sourasky Medical Center, Tel Aviv University, Tel Aviv-Yafo, Israel; 30 Endocrinology Department, Germans Trias i Pujol Hospital and Research Institute, CIBERER Unit 747, Universitat Internacional de Catalunya, Barcelona, Spain; 31 Endocrinology & Nutrition Department, Hospital Universitario de Santiago de Compostela, Santiago de Compostela, Spain; 32 Department of Life Science, Health, and Health Professions, Link Campus University, Rome, Italy; 33 Department of Endocrine and Metabolic Diseases, IRCCS Istituto Auxologico Italiano, Milan, Italy; 34 Physiologie et Physiopathologie Endocriniennes, Reference Center for Rare Pituitary Diseases (CRMR HYPO), Université Paris-Saclay, Inserm, Assistance Publique-Hôpitaux de Paris, Hôpital Bicêtre, Le Kremlin- Bicêtre, France; 35 AP-HM, Aix Marseille Univ, INSERM, MMG, MarMaRa, Marseille, France; Reference Center for Rare Pituitary Diseases (CRMR HYPO), La Conception University Hospital, Marseille, France; 36 University Hospitals Birmingham NHS Foundation Trust, Birmingham, United Kingdom; 37 Santiago de Compostela University, IDIS-Complejo Hospitalario Universitario de Santiago (CHUS), CIBERobn, Instituto Salud Carlos III, Santiago de Compostela, Spain; 38 Endocrinology Unit, Department of Clinical Medicine and Surgery, Federico II University, Napoli, Italy; 39 Department of Neurosurgery and Gamma Knife Radiosurgery, Università Vita-Salute San Raffaele, IRCCS Ospedale San Raffaele, Milan, Italy; 40 Department of Medicine, Section Endocrinology, Erasmus MC, Rotterdam, Netherlands; 41 Department of Endocrinology & Nutrition, Germans Trias i Pujol University Hospital, Badalona, Spain and CIBER-ER, ISCIII, Badalona, Spain; 42 Department of Endocrinology and Metabolism Disease, School of Medicine, Recep Tayyip Erdoğan University, Rize, Turkey; 43 Cedars-Sinai Medical Center, Los Angeles, CA USA

**Keywords:** Pituitary adenoma, Growth hormone, IGF1, Somatostatin receptor ligand, GH receptor antagonist, Transpenoidal surgery, Diabetes, Arthritis, Acral changes, Hypertension

## Abstract

The 16th Acromegaly Consensus Conference in September 2024 updated recommendations on diagnosis and treatment of acromegaly comorbidities. Since the 2020 acromegaly comorbidity management guideline was published, new evidence has emerged on novel and known comorbidities and new treatment approaches. Forty-three experts in the management of acromegaly reviewed the current literature and assessed changes in clinical practice standards and management. Current outcome goals were considered and updated, with a focus on the impact of current and emerging treatments of these comorbidities. Participants assessed factors that determine pharmacological choices, as well as use of specific agents in the management of the most relevant acromegaly comorbidities. We present consensus recommendations highlighting optimization of evidence-based acromegaly comorbidities management.

## Introduction

Acromegaly arises from autonomous growth hormone (GH) hypersecretion leading to excess circulating insulin-like growth factor 1 (IGF-I) concentrations, causing adverse effects on peripheral organs and physiologic processes [1, 2]. Patients commonly experience abnormal growth of bone and soft tissue, dysregulated glucose and lipid metabolism with increased risk for cardiovascular disease [3, 4], and consequent increased mortality risk [5]. The main treatment goal is to control excessive IGF-I concentrations, as well as signs and symptoms of the disease [6–9]. Early diagnosis and patient-centered management of comorbidities are important for optimal long-term outcomes.

The Acromegaly Consensus Group published the first set of recommendations on diagnosis and treatment of disease complications in 2003 [10, 11] and updated them in 2013 [12], and 2020 [13]. Given development of new management protocols [8], and conceptualization of the Pituitary Tumor Center of Excellence (PTCOE) [14], the field has focused on definition and optimization of management strategies for comorbidities and disease-related sequelae [15]. In September 2024, 43 experts in acromegaly management reviewed the literature and critically evaluated novel research findings and changes in clinical practice standards and opinion since the 2020 consensus. Updated recommendations focused on cardiovascular, respiratory, metabolic, and oncologic comorbidities, and bone and joint disorders, as well as the impact of this disease on kidney function, quality of life (QoL) and mortality.

## Materials and methods

**Fig. 1:**
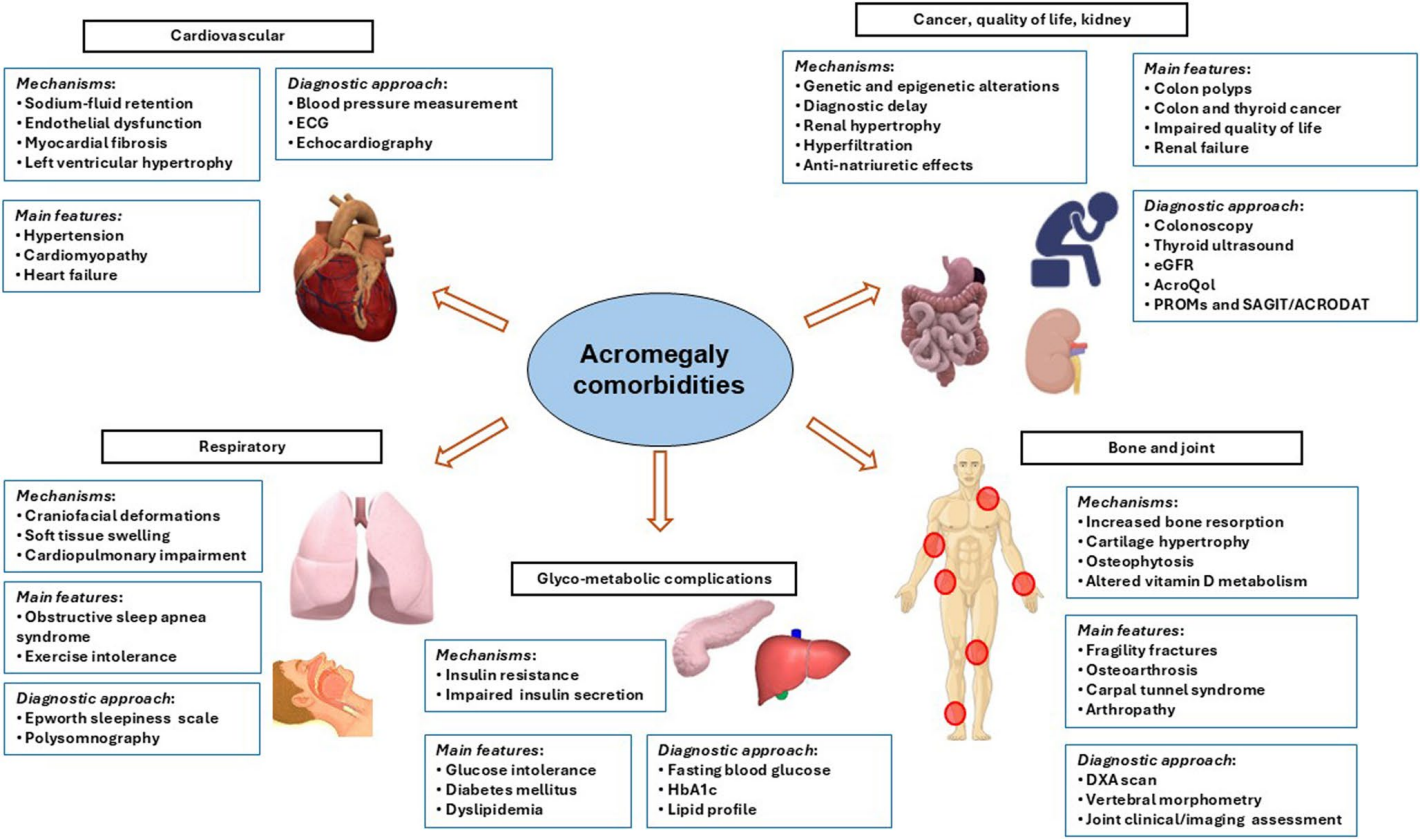
Diagnostic approach and main underlying mechanisms and clinical features of acromegaly comorbidities

Forty-three worldwide recognized experts in acromegaly management were assigned specific topics by the Consensus Co-Chairs (SM and AG) related to comorbidities and conducted comprehensive literature searches for English-language papers published between March 2018 and March 2024. In preparation of the manuscript, publications until March 2025 were also subsequently reviewed and included. Participants were selected based on their recognized expertise in the field as reflected by peer-reviewed publication records. Search terms included “acromegaly” and “comorbidities” as well as terms associated with each respective topic covered. After presentations to the entire group, parallel breakout sessions discussed current practice and recommendations, before providing summary reports back to the whole group. Consensus recommendations were based on all presentations and discussions, and participants voted on each recommendation. Divergent opinions were reconciled by voting, and all participants approved final statements included in the manuscript. Recommendations were graded using the Grading of Recommendations Assessment, Development and Evaluation system (GRADE; Table 1) [16, 17]. After the meeting, members of the Scientific Committee graded both the quality of the supporting evidence and consensus recommendations using the GRADE system. Evidence strength was graded as very low quality (VLQ), low quality (LQ), moderate quality (MQ), or high quality (HQ) [8]. Introductory sentences providing background evidence were graded with VLQ if based on expert opinion supported by one or few small uncontrolled studies; with LQ if supported by large series of small uncontrolled studies; with MQ if supported by one or few large uncontrolled studies or meta-analyses; with HQ if supported by controlled studies or large series of large uncontrolled studies with sufficiently long follow-up. Consensus recommendations were classified as discretionary (DR) if based on VLQ or LQ evidence and as strong (SR) if based on MQ or HQ evidence. Recommendations were initially discussed by each topic’s work-subgroup discussion and outcomes presented to the entire group for further discussion and consensus finalization [9]. Changes in current vs. 2020 key recommendations are summarized in Table 2. Main underlying mechanisms and clinical features as well as diagnostic approach for acromegaly comorbidities are reported in Fig. 1.

**Table 1:** Grading of evidence and recommendations

Evidence	Recommendations
• Very low quality (VLQ): expert opinion supported by 1 or a few small uncontrolled studies • Low quality (LQ): supported by large series of small uncontrolled studies • Moderate quality (MQ): supported by 1 or a few large uncontrolled studies or meta-analyses • High quality (HQ): supported by controlled studies or large series of large uncontrolled studies with sufficiently long follow-up	• Discretionary recommendation (DR): based on VLQ or LQ evidence • Strong recommendation (SR): based on MQ or HQ evidence

**Table 2:** Key changes from the 2020 to the 2024 consensus recommendations for diagnosis and follow-up of acromegaly comorbidities

Assessment	Frequency	
	2020	2024
**Cardiovascular and respiratory disorders**		
Blood pressure measurement	At baseline and every 6 months or upon change of antihypertensive treatment	Not modified ABMP is recommended over office measurements
Echocardiography	Annually, if abnormal	At diagnosis, annually if abnormal
CMRI	Not addressed	Consider at diagnosis, individualized follow-up
Electrocardiography	Annually, if abnormal	At diagnosis, individualized follow-up
Holter electrocardiography	Not addressed	In patients at high risk (history of syncope, abnormal ECG, structural abnormalities, heart failure)
Routine exercise program	Not addressed	In all patients without contraindication
Epworth scale and/or Polysomnography	Baseline or before surgery if OSAS is suspected	At diagnosis, individualized regular monitoring
**Endocrine and metabolic disorders**		
Fasting blood glucose, HbA1c	Fasting blood glucose every 6 months, particularly in uncontrolled disease and during SRL therapy; HbA1c every 6 months if diabetes or prediabetes is present	Glycemic status assessment every 6 months, more frequent (3 months) if out of target and/or after treatment modification
Lipid profile	Follow general guidelines	At diagnosis, every 8 ± 4 weeks after the treatment, annually after the achievement of target. Follow general guidelines for the treatment.
MASLD	Not addressed	Assessment in patients with risk factors (GHD)
Body Composition	Not addressed	Consider at diagnosis with DXA scans or BIA, individualized follow-up
Total testosterone, SHBG, and PRL (males)	Annually, consider testing free testosterone if there are doubts in interpretation of total testosterone	Not addressed
LH, FSH, 17β-estradiol, and PRL (females)	Annually, in premenopausal females with menstrual dysfunction and when pregnancy is desired	Not addressed
Serum free T4	Annually	Not addressed
Serum 8–9 am cortisol	If central adrenal insufficiency is suspected; cosyntropin stimulation test if serum cortisol is low	Not addressed
**Musculoskeletal disorders**		
Bone complications		
DXA	Every 2 years particularly if osteopenia/osteoporosis is present	Not modified
Vertebral morphometry on thoracic x-ray, thoracic and lumbar spine x-ray	Annually, particularly if history of vertebral fracture, decrease in BMD, kyphosis, symptoms of vertebral fracture, untreated hypogonadism, and no biochemical control of acromegaly	Not modified
TBS	Not addressed	When available, particularly in patients with high risk factors
Arthropathy	Early assessment is recommended	Early assessment of joints with specific questionnaires (e.g. WOMAC) and radiologic tools (e.g. X-Ray, MRI)
**Other comorbidities**		
Cancer		
Colonoscopy	Every 10 years; more frequently if IGF-I remains persistently elevated or if abnormal colonoscopy or family history of colon cancer	At diagnosis. During follow-up according to country-specific guidelines
Thyroid ultrasound	In patients with palpable thyroid nodules and/or with risk factors of thyroid cancer	Not modified
Kidney		
GFR	Not addressed	At diagnosis, annually thereafter
Renal ultrasound	Not addressed	Individualized at diagnosis and follow-up
Water and electrolyte balance	Not addressed	At diagnosis, annually thereafter
**Quality of life**		
AcroQoL	Annually	Not modified
Combination of PROMs (AcroQoL) and SAGIT/ACRODAT	Not addressed	Annually

*Abbreviations*: *BMD* bone mineral density, *DXA* dual-energy x-ray absorptiometry, *OSAS* obstructive sleep apnea syndrome, *PRL* prolactin, *SHBG* sex hormone binding globulin, *SRL* somatostatin receptor ligand, *GHD* growth hormone deficiency, *BIA* bioelectrical impedance analysis, *ABPM* ambulatory blood pressure monitoring, *GFR* glomerular filtration rate, *MRI* magnetic resonance imaging, *CMRI* cardiac MRI, *ECG* electrocardiography, *MASLD* metabolic dysfunction-associated steatotic liver disease, *TBS* trabecular bone score, *QoL* quality of life, *PROMs *patient-reported outcome measures, *HbA1c* hemoglobin A1C, *LH* Luteinizing Hormone, *FSH *follicle stimulating hormone, *T4 *thyroxine, *IGF-I* insulin-like growth factor 1, *WOMAC* Western Ontario and McMaster universities osteoarthritis index

### Determinants of acromegaly complications

Acromegaly signs and symptoms as well as comorbidities are mediated directly by either GH or IGF-I (HQ) (Fig. 1). Non-IGF-I mediated actions of GH include lipolysis, gluconeogenesis and glycogenolysis, together with decreased tissue glucose uptake and glucose oxidation [18] (MQ). GH directly inhibits insulin receptor signaling, attenuating suppression of hepatic glucose production and peripheral glucose utilization [18]. Stimulation of lipolysis by GH further hampers insulin sensitivity, impairs beta-cell function, and reduces whole-body fat [19]. GH also acts directly on epithelial channels in the distal nephron causing water and sodium retention (MQ) [20].

Glucose tolerance improves following successful surgical resection of the adenoma or with first-generation somatostatin receptor ligands (fg-SRLs), suggesting that reversal of GH excess ameliorates insulin sensitivity (HQ) [21]. Patients with hypertension and diabetes mellitus have more severely impaired cardiac performance than those without (MQ) [22]. GH excess reversibly reduces fat mass, increases extracellular water (ECW), and increases resting energy expenditure. GH promotes nephron sodium retain leading to expanded ECW [23] with soft tissue swelling, including tongue edema, with characteristic facial dysmorphism (MQ). Upper airway swelling may result in snoring or sleep apnea which improves following treatment (MQ). Myocardial edema may contribute to cardiomyopathy [24].

Prevalence and severity of disease complications arise from cumulative exposure to high IGF-I (MQ). Circulating IGF-I is the best marker of GH action, and its measurement by a validated assay with robust age-related reference ranges is the best biochemical marker of disease activity (MQ). IGF-I exerts anti-lipolytic effects on adipose tissue similar to that of insulin and opposite to GH effects (HQ) [25, 26]. Insulin sensitivity is reduced by GH while IGF-I increases insulin sensitivity (MQ) [27]. GH normalization is associated with improved life expectancy and ameliorates obstructive sleep apnea syndrome (OSAS) (MQ) [3]. IGF-I normalization leads to blood pressure (BP) lowering (in hypertensive patients), decrease in fasting plasma glucose (FPG), homeostatic model assessment for insulin resistance (HOMA-IR) and HOMA-B and increased HDL- cholesterol (C) but no change in LDL-C [28].

Successful surgery can reduce BP in patients with hypertension (LQ). FPG and HOMA-IR decreased following surgery since lowering GH per se improves insulin sensitivity [28]. SRLs may have overall neutral effects, as their suppression of insulin secretion may counterbalance the improvements in insulin sensitivity due to acromegaly control (MQ) [29]. IGF-I promotes vascular relaxation through modulation of sodium/potassium ATPase activity [30] with improved cardiac output, stroke volume, contractility, and ejection fraction (MQ). IGF-I receptors (IGF-IR) are abundantly expressed in cardiomyocytes, vascular endothelial cells, and smooth muscle cells [31], leading to vasodilatory effects primarily by enhancing endothelial nitric oxide synthase activity. IGF-I also improves lipid profile, lowers insulin concentrations, and increases insulin sensitivity [32].

GH stimulates transepithelial sodium transport in the distal nephron through activation of epithelial sodium channels (ENaC) in the collecting duct (MQ). IGF-I enhances sodium reabsorption by activating serum- and glucocorticoid-induced kinase 1 (Sgk1) increasing the membrane residency of active ENaCs. IGF-I may also cooperate with GH to regulate ENaC gene expression.

IGF-I is essential for differentiation and maturation of growth plate chondrocytes, promoting linear bone growth and osteoblastogenesis, enhancing collagen production, and supporting bone matrix mineralization, thereby contributing to bone formation. In genetically modified mouse models circulating IGF-I is critical for cortical bone integrity, while locally produced skeletal IGF-I is required for the maintenance of cancellous (trabecular) bone. Together, these findings underscore the dual systemic and local roles of IGF-I in preserving both bone architecture and function (MQ) [33–35]. On the other hand, GH exerts direct anabolic actions on bone through its receptor (GHR), independent of hepatic IGF-I production. GHR is expressed in multiple skeletal compartments, including growth plate chondrocytes, osteoblasts, and mesenchymal stromal cells (MSCs). GH binding to GHR activates intracellular signaling cascades, primarily the JAK-STAT signaling pathway (especially STAT5), but also MAPK and PI3K/Akt pathway which promote chondrocyte proliferation, osteoblast differentiation, and anti-apoptotic effects in bone-forming cells. In animal models, local GH administration stimulates longitudinal bone growth even in the absence of IGF-I receptor activity, confirming an IGF-independent effect. GH directly increases bone formation parameters, enhances mineral apposition, and maintains cortical and trabecular bone mass. At the marrow level, GH favors osteoblastogenesis over adipogenesis by modulating MSC commitment and inhibiting adipogenic differentiation. These direct skeletal actions are essential for bone growth, microarchitectural integrity, and homeostasis throughout life [36].

## Cardiorespiratory complications

### Consensus recommendations

*Diagnosis*:

- *At diagnosis*,* an ECG assessment is recommended in all patients (SR)*.
- *Ambulatory Blood Pressure Monitoring (ABPM) is recommended for the diagnosis of hypertension (DR)*.
- *Assessment of cardiac function and morphology should be performed*,* at diagnosis including an echocardiography (DR)*.
- *Specialized imaging techniques (e.g.*,* speckled tracking echocardiography*,* CMRi) are not currently recommended (DR)*.
- *In newly diagnosed patients*,* polysomnography should be systematically performed at diagnosis (even in the absence of symptoms) to properly identify and manage obstructive sleep apnea syndrome (OSAS) (DR)*.

*Therapy*:

- *Patients with congestive heart failure require adequate therapy*,* and prevention of cardiac arrhythmia (SR)*.
- *Hypertension may persist despite acromegaly control*,* and current guidelines for the general population should be followed (DR)*.
- *Regardless of acromegaly control*,* treatment of OSAS should be instituted (DR)*.
- *Continuous positive airway pressure with a specially fitted mask is recommended for patients with OSAS and atypical facial morphometry (DR)*.
- *An individualized exercise program to improve exercise tolerance should be considered if there are no contraindications (DR)*.

*Follow up*:

- *Follow-up echocardiography should be performed as clinically indicated (DR)*.
- *ABPM is recommended for following patients with hypertension (DR)*.
- *Patients at risk for arrythmias (history of syncope*,* abnormal ECG*,* structural abnormalities*,* heart failure) may benefit from ambulatory ECG monitoring (DR)*.
- *Regular monitoring with polysomnography is recommended because OSAS may persist or worsen despite appropriate acromegaly therapy (DR)*.

Mortality rates have decreased in well-controlled acromegaly closely approximating that of the general population (HQ) [37, 38]. Cardiovascular disease is an important cause of mortality in patients with acromegaly despite the recent shift to age-related cancers as the leading cause of death (MQ) [37, 39–43]. It remains unclear how much of this shift is due to improved treatment of acromegaly versus overall improved cardiac care.

### Hypertension

The prevalence of hypertension is estimated at approximately 40% [4, 44–47], reaching up to 60% in some series, with higher rates in patients with biochemically active acromegaly and may occur at a younger age than in the general population (HQ) [48, 49]. Hypertension significantly increases mortality, particularly when accompanied by cardiovascular disease highlighting the critical importance of prompt screening and management of cardiovascular complications to reduce morbidity and mortality (HQ) [49]. Hypertension is related to insulin resistance, expansion of ECW, increased peripheral vascular resistance, and sleep apnea [45], and these effects may not be fully reversible. Importantly, hypertension may persist despite biochemical remission [50], particularly in older patients [45, 48].

The 24 h-ambulatory blood pressure monitoring (ABPM) is recommended over office measurements for the diagnosis and follow-up of hypertension (DR) [51, 52]. Sodium overload may be prevented by the ENaC blocker amiloride and other diuretics if needed [53]. Surgical resection of the GH-secreting adenoma was demonstrated to reduce BP after 3- and 6-months post-surgery (MQ). 24 h-ABPM also confirmed a significant decrease in BP, with restoration of normal circadian BP rhythms (MQ). The benefits were more evident in patients with controlled IGF-1 concentrations, and not in those with uncontrolled acromegaly [45, 54–57]. In some studies, successful control of GH and IGF-I concentrations with fg-SRLs was associated with improved control of hypertension (LQ) [45, 54, 58]. However, a meta-analysis of 16 studies including 190 patients did not demonstrate direct effect of fg-SRLs on BP [59]. These findings suggest that the antihypertensive benefit sometimes observed with fg-SRLs is likely an indirect consequence of GH and IGF-I normalization, rather than a direct pharmacodynamic effect of the drugs themselves (LQ). Improved BP following pegvisomant treatment could support a direct pathogenic role of GH/IGF-I excess in hypertension development (LQ) [49].

### Cardiomyopathy and heart failure

Cardiomyopathy has been considered a hallmark morbidity of acromegaly due to the impact of GH and IGF-I on the heart (HQ). GH enhances cardiomyocyte sensitivity and content of myofilament calcium, L-type calcium channels, and collagen deposition. In addition, cardiac hypertrophy might be induced by pressure or volume overload [4].

Cardiomyopathy may occur early in the course of the disease with specific cardiac alterations, mainly characterized by a reversible hyperkinetic left ventricle (LV) in the first stage; followed by a second stage of progressive hypertrophy leading to diastolic dysfunction and, a third stage of both diastolic and systolic dysfunction (MQ). Cardiomyopathy occurs independently of hypertension and diabetes but worsens with disease duration and other cardiovascular risk factors (HQ) [60–62]. The prevalence of LV hypertrophy (LVH) ranged between 11% and 78% and diastolic dysfunction between 11% and 58% of patients with this wide variation in percentages among the series due to different study design or overestimation of LVH with echocardiography [4]. Recently, speckle tracking echocardiography (STE) and cardiac magnetic resonance imaging (CMRi) have been used for definition of cardiac abnormalities (VLQ). However, there is currently insufficient research on STE to explore myocardial strain parameters in acromegaly [63]. Non-invasive CMRi is not routinely used in clinical practice but it may have advantages in the assessment of cardiac abnormalities with respect to echocardiography. In fact, it revealed lower frequency (5%) of LVH supporting overestimation of LV hypertrophy with echocardiography [64]. CMRi with late gadolinium enhancement enables direct visualization of myocardial fibrosis. T1 mapping and ECV quantification are advanced CMRi techniques allowing for precise, non-invasive assessment of myocardial tissue. T1 mapping correlates with myocardial collagen content, supporting their role as surrogate markers of fibrosis [64]. A multicenter case-control study using CMRi imaging demonstrated that compared to age-, sex-, and body mass index (BMI)-matched controls with similar cardiometabolic risk profiles, acromegaly patients exhibited increased biventricular volumes even with biochemical control, higher left ventricular mass (LVM), and reduced right ventricular systolic function [65].

Structural heart disease may improve with biochemical control of the disease (MQ). Surgical adenoma resection leads to rapid normalization of GH and IGF-I and reduced LVM, improved diastolic function, and resulted in partial or complete reversal of LVH (MQ). Postoperatively, BP normalization, reduced heart rate and increased left ventricular ejection fraction (LVEF) during exercise have been demonstrated [4, 66]. A meta-analysis showed that both lanreotide and octreotide reduced LVM in patients achieving disease control [59], with improved mitral inflow and better diastolic function (HQ). Compared with surgical remission, SRL therapy similarly decreased LVM, diastolic BP, with the added benefit of a modest increase in LVEF, despite normal baseline values (VLQ) [58]. Pegvisomant was associated with gradual improvements in cardiac comorbidities alongside suppressed IGF-I concentrations (MQ). Systolic dysfunction (defined as LVEF < 60%) improved with pegvisomant, and LVEF normalized in patients with hyperkinetic syndrome (LVEF > 70%). LVH also improved with pegvisomant treatment, as demonstrated by a decrease in LVM index in patients with the most severe LVH [67].

Baseline assessment of cardiac function and morphology should be conducted at diagnosis and individualized based on clinical characteristics. Prompt achievement of biochemical control is a primary therapeutic goal. Although treatment of acromegaly improves cardiac function in the short term, it probably has little or no effect on longstanding myocardial hypertrophy, ventricular dilation, or the long-term prognosis in patients with congestive heart failure, as myocardial damage is irreversible at this late stage of cardiomyopathy [68].

The pathogenesis of arrhythmias might be related to structural cardiomyopathy (LQ) [61]. The prevalence varies reaching up to 40% in older studies [69]. In contrast, a prospective study showed no sustained arrhythmias [70]. Notably, a recent cohort study found an increased atrial fibrillation risk during the first four years after diagnosis of acromegaly, although this association lost significance over time [46]. Patients with acromegaly may rarely exhibit prolonged QT interval [71]. Elevated beat-to-beat short-term QT interval variability and higher prevalence of late potentials may underly arrythmia [72, 73]. Treatment selection of acromegaly should consider potential effects on QT interval i.e., the possible risk of QT interval prolongation with pasireotide [74, 75]. Therefore, QT interval monitoring before and during treatment is advisable [76]. Also, bradycardia is commonly observed with all SRLs [59, 77]. Long-term treatment with pegvisomant could improve rhythm abnormalities [78]. In patients at risk for arrythmias (history of syncope, abnormal baseline ECG, structural abnormalities, heart failure) Holter ambulatory ECG monitoring may be indicated (DR).

Heart failure is rare now in acromegaly, ranging between 1% and 4% [46, 79] and is likely influenced by disease duration and severity, the presence of cardiovascular risk factors and family history (LQ) [80]. Valvular dysfunction is reported in up to 86% of patients, but very rarely of clinical significance, most commonly affecting the mitral and aortic valves, and is irreversible despite successful biochemical control [61].

### Obstructive sleep apnea syndrome

The prevalence of OSAS is very high in acromegaly, in up to 80% of newly diagnosed patients. OSAS results mainly from characteristic pharyngeal soft tissue swelling (HQ) (81). OSAS impact on multiple comorbidities is significant (HQ), i.e. representing an independent risk factor for ischemic heart disease, arrhythmia, and cardiomyopathy (HQ) (82, 83). Poor sleep quality adversely affects QoL, and cognitive function (84). Acromegaly treatment improves OSAS in most patients, but it might persist even in controlled patients, requiring close monitoring (MQ) (85-89). Preoperative treatment with SRLs may improve pharyngeal swelling and facilitate intubation during surgery. Patients should undergo OSAS assessment at diagnosis, with a thorough questioning of spouse/partner, and use of a sleep questionnaire, such as the Epworth sleepiness scale or the STOP-BANG questionnaire, although it is important to be aware that such questionnaires may underestimate the presence or severity of sleep disordered breathing (90). Accordingly, polysomnography or respiratory polygraphy should be systematically performed at baseline (even in the absence of symptoms), with regular monitoring recommended thereafter because OSAS may persist or worsen despite appropriate acromegaly therapy (DR) (91, 92). Continuous positive airway pressure with a specially fitted mask may be necessary for patients with atypical facial morphometry (DR).

### Exercise intolerance

Patients with acromegaly may have decreased exercise tolerance due to cardiopulmonary impairment, muscle weakness, physical changes and hypopituitarism (LQ) [62, 93].

Studies found decreased LVEF at peak exercise, and an inverse correlation with both disease duration and age, while positively correlating with peak left ventricular filling rate [94]. In patients with preserved LV systolic function at rest, the LVEF response during physical exercise may still be impaired, likely due to underlying LV diastolic dysfunction. These findings highlight the relevance of diastolic abnormalities in limiting exercise capacity in acromegaly (VLQ) [95]. Respiratory complications, including altered craniofacial bones and soft tissues, respiratory mucosa, and cartilage, as well as increased lung volume, modified rib cage geometry, reduced lung elasticity, and increased distensibility contribute to impaired ventilatory efficiency (LQ). Patients often exhibit inadequate ventilatory response to increased exercise demand, with reduced workload capacity at both the anaerobic threshold and peak exertion [62]. Cardiopulmonary impairment is characterized by decreased maximal oxygen consumption (VO₂max) [62, 81]. Aerobic capacity typically reaches only 60–85% of age- and sex-adjusted normative values [96].

Decrease of GH and IGF-I concentrations with fg-SRLs was shown to improve exercise time, cardiac and ventilatory performance (LQ) [97, 98]. Following octreotide, increases in workload and oxygen consumption are observed at both anaerobic threshold and maximal effort. Importantly, after treatment, cardiopulmonary performance is partially restored [97]. Octreotide decreased GH and IGF-I concentrations and improved exercise performance, with an increase in exercise time until exhaustion or reaching age-related predicted target heart rate is reached during treadmill testing [99]. Reduced heart rate at peak exercise with octreotide was observed exclusively in younger patients. Normalization of LVEF response to exercise was achieved in 69.2% of patients overall, with a higher prevalence in younger (80%) compared to middle-aged patients (50%) [98]. Treatment with octreotide for 6 months led to significant improvements in ventilation threshold (VeT) and perceived vigor, accompanied by a modest increase in VO₂max as assessed during treadmill testing. Reduced serum IGF-I concentrations correlated with changes in VeT and subjective vigor scores. Taken together, these findings reinforce that maintaining physiologic GH/IGF-I concentrations is crucial for preserving optimal physical function (MQ) [100]. Short-term regular exercise improves cardiopulmonary function and exercise time and reduces heart rate during warm-up [101].

Skeletal muscle is a primary target tissue for GH and IGF-I and increased fatty infiltration (ectopic fatty deposition) and reduced muscle performance are seen in acromegaly (LQ). These alterations appear to be further exacerbated by active disease [102]. A recent study reported a high prevalence of sarcopenic obesity among patients with acromegaly, which may contribute to impaired exercise performance [103].

## Endocrine and metabolic disorders

### Consensus recommendations

*Diagnosis*:

- *Glucose metabolism should be investigated at diagnosis and during follow-up with fasting glucose concentrations and HbA1c assessment (SR)*.
- *Since BMI does not reflect body composition alterations*,* evaluation should be undertaken by DXA or multifrequency bioimpedance at baseline and during disease treatment if available (DR)*.

*Therapy*:

- *Alterations in glucose metabolism and diabetes should be restored with acromegaly disease control (DR)*.
- *Treatment of impaired glucose tolerance and diabetes mellitus should entail biochemical control of acromegaly while following the general principles of management of diabetes mellitus (DR)*.
- *A personalized*,* shared approach should be used to select glucose-lowering medications*,* considering effectiveness*,* comorbidities*,* side effects*,* and patient preference (DR)*.
- *Metformin is the first treatment choice (DR)*.
- *Risks and benefits of SGLT2 inhibitors should be balanced in uncontrolled acromegaly between the cardiovascular and renal protection and the potential risk of euglycemic ketoacidosis (DR)*.
- *GLP-1 receptor agonists may be used in selected patients offering benefits beyond glycemic control*,* including improved cardiovascular and renal outcomes (DR)*.
- *Correction of dyslipidemia is recommended by specific treatment*,* due to detrimental effects on cardiovascular co-morbidities (DR)*.

*Follow up*:

- *Patients with metabolic syndrome require increased awareness and intensive treatment of dyslipidemia*,* insulin resistance*,* prediabetes*,* all typically observed in acromegaly (DR)*.
- *GH deficiency (GHD) may be associated with an increased risk of metabolic dysfunction-associated steatotic liver disease and therefore overtreatment of acromegaly causing GHD should be avoided. If GHD develops*,* GH replacement therapy should be considered (DR)*.
- *Patients with GHD may require more frequent liver function monitoring than recently suggested by MASLD guidelines (DR)*.

### Diabetes mellitus

Incidence of diabetes mellitus is increased, potentially affecting survival (HQ). Altered glucose metabolism is a common complication, with a reported diabetes mellitus prevalence of 30%, typically present at the time of diagnosis [104–107]. Patients with acromegaly have an increased risk of developing diabetes mellitus (HR: 4.0; 95% CI: 2.7–5.8), being most pronounced in the three years preceding diagnosis [79], primarily due to GH-induced insulin resistance. GH also directly impairs insulin signaling and stimulates lipolysis, leading to elevated free fatty acids (FFAs) that enhance hepatic gluconeogenesis and reduce glucose uptake [108]. Diabetes significantly increases mortality, with a 60% higher overall risk and a 2-fold increase in cardiovascular mortality (HQ) [108]. Patients should be evaluated for glycemic status both at diagnosis and during the follow-up using FPG and hemoglobin A1c (HbA1c) [13, 109, 110].

To optimize outcomes, hyperglycemia should be closely managed (Table 2) and targeting biochemical acromegaly remission should be the fist-step (DR) [107]. Improvement is seen after pituitary surgery, especially in patients with preserved β-cell function (HQ) [111–113]. After surgical remission, and in patients with preserved anterior pituitary function, a multicenter study reported remission of diabetes in 20% of patients; especially in the elderly [113]. Octreotide and lanreotide rarely impair glucose homeostasis, and generally have an overall neutral effect on FPG and HbA1c [114], mainly related to disease control, however glucose metabolism monitoring is essential, with emphasis on post prandial glucose (MQ) [115]. In contrast, pasireotide may have a detrimental effect on glucose metabolism, driven by its suppressive effect on insulin and incretin secretion [116], therefore it is not recommended in patients with poor glycemic control particularly if not treated with insulin (MQ) [8, 117]. The side effect usually manifests early during treatment and is usually manageable with appropriate treatment with incretin-based therapies and is reversible upon discontinuation [118–121]. Pegvisomant improves glucose metabolism (MQ) with a positive impact on insulin resistance, partially independently of disease control and may be considered as a valuable option either as monotherapy or in combination with SRLs, in patients with severely impaired glucose metabolism (MQ) [8, 122].

A personalized, shared decision-making approach should be used to select glucose-lowering medications, considering effectiveness, comorbidities, side effects, and patient preference (DR) [123]. The initial step for mild glucose abnormalities is lifestyle interventions, diet, physical activity and weight loss (MQ). Metformin is the first-choice medical therapy if not contraindicated (LQ). Sodium–glucose cotransporter 2 (SGLT2) inhibitors may exert favorable effects on acromegaly disease control and might be useful, particularly in patients with cardiovascular and renal comorbidities, although they must be used with caution due to the risk of ketoacidosis, especially in patient with active acromegaly and insufficient pancreatic β-cell reserve (LQ) [123–125]. Decreased insulin secretion leads to reduced paracrine inhibition of glucagon release, further promoting ketogenesis. In the context of acromegaly, GH excess independently promotes lipolysis, which can further drive ketogenesis. Despite this theoretical risk, diabetic ketoacidosis has rarely been reported during SGLT2 inhibitor treatment. A recent study showed no reported adverse effects related to these medications which support the cautious use of SGLT2 inhibitors as a potential treatment option in patients with well-controlled acromegaly [107]. Pasireotide treatment could be a contraindication for SGLT2 inhibitor use due to its inhibitory effects on pancreatic β-cell function, which may exacerbate hyperglycemia and increase the risk of diabetic ketoacidosis (VLQ) [116]. Additionally, in patients treated with SGLT2 inhibitor osmotic diuresis might be enhanced resulting in impairment of renal function which should initially be closely monitored, particularly in older patients and those receiving GH receptor antagonists, as this class of drugs opposes the sodium-retaining effects of GH [24, 118]. Dual GIP/GLP-1 receptor agonist should be used with caution, since GIP has been implicated in paradoxical GH response glucose being clinical impact of this not yet known (VLQ) [126].

### Dyslipidemia

Acromegaly is often associated with an atherogenic dyslipidemia patterns (MQ) which should be checked at baseline and appropriately monitored during follow-up (Table 2). The prevalence of dyslipidemia is 30% [105], potentially driven by GH excess, glucose intolerance, diabetes mellitus, and metabolic syndrome (MQ). Acromegaly is also associated with mild to moderate hypertriglyceridemia. Compared to healthy controls, acromegaly patients have elevated concentrations of triglycerides (TG) and lipoprotein(a) [Lp(a)], with reduced total cholesterol (TC), HDL-C, LDL-C, apolipoprotein A1, and apolipoprotein B in the acromegaly cohort compared to controls [127]. Elevated Lp(a) concentrations may contribute to atherosclerotic cardiovascular disease [128]. Insulin resistance impacts the metabolism of TGs, HDL-C, LDL-C, and very low-density lipoprotein cholesterol [129]. Elevated concentrations of FFAs due to increased lipolysis, the most prominent metabolic effect of GH, increase the risk of development of insulin resistance [130, 131].

Acromegaly treatment has inconsistent effects on lipid profile (LQ). Surgery generally lowers TGs, and LDL-C and increases HDL-C [111]. Octreotide and lanreotide decrease TGs and raise HDL-C [132, 133]. LDL-C may increase with pegvisomant treatment whereas LP(a) concentrations decrease after surgery and SRL treatment [28]. The diagnosis, treatment, and management of dyslipidemia to prevent cardiovascular disease should adhere the current evidence-based lipid guidelines [134–136].

### Metabolic dysfunction-associated steatotic liver disease (MASLD)

MASLD may have relevant cardiovascular implications in patients with acromegaly (VLQ). GH promotes lipolysis and mobilizes FFAs into the circulation while also enhancing FFA β-oxidation, expected to reduce hepatic lipid accumulation (HQ). GH suppresses hepatic de novo lipogenesis. Interestingly, despite significant insulin resistance in acromegaly, hepatic lipid concentrations are reduced [137]. This paradox may be explained by increased hepatic mitochondrial activity [138] or decreased FGF21 [139, 140]. Nevertheless, genetic predisposition driven by the PNPLA3 susceptibility allele has been identified as a contributing risk factor for hepatic steatosis in acromegaly [141]. Moreover, combination with pegvisomant and a reduced-dose SRL, has been associated with increased intrahepatic lipid compared to SRL in monotherapy [142]. In contrast, GH deficiency (GHD) is strongly associated with an increased prevalence of MASLD [143, 144]. Therefore, patients with GHD may require more frequent liver function monitoring than recently suggested by MASLD guidelines (DR) [145].

### Body composition

GH and IGF-I regulate body composition (HQ). Patients with acromegaly exhibit fluid retention and low-fat mass and a specific type of lipodystrophy (MQ) which is characterized by reduced adiposity despite significant insulin resistance (LQ). Visceral adipose tissue (VAT) and subcutaneous adipose tissue are decreased, while intermuscular adipose tissue increases [146]. Recently, a high prevalence of sarcopenic obesity has been demonstrated, although one study reported no differences in sarcopenic obesity in patients with acromegaly compared to control group [103, 147]. After successful somatotroph adenoma resection, central adiposity increases, including VAT and intrahepatic lipid, while insulin sensitivity improves [148]. VAT increases in the short-term treatment with pegvisomant, however, this finding plateaus during long-term treatment [149].

Lean body mass (LBM), as estimated by dual-energy x-ray absorptiometry (DXA) is typically increased in acromegaly, likely due to excess ECW compartment rather than increased skeletal muscle (MQ) [15]. Similarly, reduced LBM during octreotide treatment have been attributed to decreases in soft tissue fluid rather than true muscle loss [150]. Skeletal muscle mass did not change with long-term pegvisomant [149]. Measuring BMI is of limited diagnostic utility due to the unique pattern of acromegaly body composition changes. Since BMI does not reflect body composition, it should be assessed with DXA or bioelectrical impedance analysis when clinically indicated at diagnosis and follow-up (DR). Skeletal muscle estimates from DXA-adjusted limb tissue strongly concurred with MRI measurements, validating the use of DXA prediction equations for assessing skeletal muscle [150]. While data on muscle mass are conflicting, higher fatty atrophy and lower muscle performance was demonstrated with a further detrimental effect of active acromegaly (VLQ) [102].

## Bone complications

### Consensus recommendations

*Diagnosis*:

- *Vertebral morphometry using spine X-ray or latero-lateral scan during DXA should be performed at diagnosis (SR)*.
- *As BMD measured by DXA and FRAX score are not reliable parameters to predict vertebral fracture (VF) risk*,* assessment of bone quality* with *DXA-derived trabecular bone score (TBS) is recommended (DR)*.
- *Vitamin D status should be evaluated at diagnosis (DR)*

*Therapy*:

- *Bone fracture preventing agents should be initiated based on clinical judgment and available guidelines (DR)*.
- *Cholecalciferol supplementation should be recommended in acromegaly patients with low vitamin D concentrations and/or high risk of fractures (DR)*.

*Follow-up*:

- *Patients without VFs should be monitored by DXA-derived BMD and TBS (DR)*
- *In patients with prevalent vertebral fractures and/or decreased BMD and/or bone quality, persistently active disease or untreated hypogonadism, monitoring with morphometry should be recommended (DR).*

### Clinical assessment

Skeletal fragility is a frequent complication of acromegaly (HQ). GH and IGF-I directly and/or indirectly regulate bone metabolism; excess concentrations lead to increased bone turnover and deteriorated trabecular and cortical bone microarchitecture, as determined by changes in biochemical markers and calcium kinetics, leading to bone loss [151–155].

Vertebral fractures (VFs) are highly prevalent in acromegaly and contribute significantly to reduced QoL (HQ). Morphometric VFs represent an early and frequent sign of impaired bone health, with a median prevalence of 40% [142]. The risk of VFs is particularly high in patients with prior VFs [140, 143], more active disease at diagnosis, greater diagnostic delay, hypogonadism, or vitamin D deficiency [144–146] (MQ). Patients with acromegaly demonstrated a significantly increased risk of hip fractures in addition to clinical VFs compared with controls. This excess fracture risk was time-dependent and became evident early during the follow-up period [156]. Preexisting diabetes mellitus, GHD, and overtreatment of secondary adrenal insufficiency and hypothyroidism are also contributory factors [154, 157–159]. VFs are related to further fracture risk, decreased survival, and lower QoL in the general population [160]. Bone mineral density (BMD) using DXA has limited value for fracture risk prediction, as VFs may occur despite normal or increased BMD due to predominant deterioration in bone quality [161, 162]. An overestimation of lumbar spine (LS) BMD in acromegaly may result from joint degenerative changes, including osteophyte formation, facet joint hypertrophy, and bone enlargement (MQ). Since VFs are often silent and underdiagnosed, their radiological screening (spine X-ray or latero-lateral scan with DXA or chest X-ray) is important at diagnosis.

Bone quality is compromised despite preserved BMD, with altered structure at trabecular and cortical components (MQ). While BMD often remains normal, TBS and 3D-SHAPER analyses consistently show lower trabecular integrity. Postsurgical remission appears to partially restore TBS, whereas pituitary radiotherapy may worsen it. High-resolution peripheral quantitative computed tomography and high-resolution cone-beam CT may show increased cortical porosity, reduced trabecular number, and impaired cortical strength, even in eugonadal or biochemically controlled patients. These changes are more pronounced in patients with VFs. Additionally, microindentation and quantitative ultrasound studies demonstrate persistent deficits in cortical bone properties and mechanical competence. Collectively, these findings support the need for advanced imaging modalities beyond BMD to assess bone fragility and suggest that fracture risk may be more accurately predicted by bone quality parameters than by BMD alone [152, 163, 164].

### Vertebral fractures monitoring

A high prevalence of radiological VFs has been reported in newly diagnosed patients supporting the notion that VFs may represent an early skeletal complication of the disease (MQ). Notably, presurgical GH concentrations predict VF risk, as assessed by morphometric vertebral evaluation highlighting the importance of incorporating VF screening into the initial diagnostic workup, especially in patients presenting with random GH concentrations > 12 ng/mL at diagnosis [165]. Incident VFs (i-VFs) occurred in 34.3% of acromegaly patients, with a diagnostic delay of > 10 years associated with a 1.5-fold increased risk of developing i-VFs [166]. Early identification of VFs can facilitate personalized GH-lowering therapies and consideration of bone-targeted treatments. A prospective longitudinal cohort study revealed VF progression in 35% of patients over a 9-year follow-up period [167]. Notably, progression occurred more frequently among patients not receiving ongoing medical therapy. Previous studies have reported VF progression in 20–25% of treated acromegaly patients during shorter follow-up periods [158, 168]. Interestingly, patients receiving SRLs, were less likely to experience VF progression in contrast with previous observations suggesting increased risk of arthropathy progression in patients treated with SRLs, highlighting a potential divergence in skeletal outcomes based on the mechanism of medical treatment [167]. Moreover, SRLs may confer bone-protective effects since they are associated with a reduced risk of hypopituitarism compared with surgical or radiation approaches [167, 169]. Evaluation of VFs at follow-up should be patient-centered guided by symptoms and back pain, prior VFs, previous diagnosis of osteoporosis, presence of diabetes mellitus, disease activity [152, 161]. Vertebral morphometry should be performed on spine X-ray or lateral scans during DXA, or using chest X-ray performed for other clinical indications if available, particularly in high-risk patients (SR). DXA BMD measurement can still have a role, as femoral neck BMD is reduced, especially in patients experiencing i-VFs [158]. In the presence of low BMD, DXA measurements should be performed every 18–24 months depending on patient characteristics, in line with most guidelines [170–172]. DXA-derived bone quality measurements may provide valuable insights for predicting fracture risk and optimizing management of skeletal complications [152]. The FRAX score calculation, an objective quantitative estimate for fracture risk, was not useful for fracture prediction in patients with acromegaly [103, 173].

Pharmacological antiosteoporosis therapy should be considered individually based on clinical and biochemical findings, due to limited data elucidating prevention of fractures in active acromegaly (LQ) [174]. Current evidence does not allow for rigorous recommendations on the choice of medical acromegaly treatment in relation to bone health. Results from a limited number of patients revealed less frequent i-VFs with pasireotide compared to pegvisomant in patients resistant to SRLs [175]. As pegvisomant alone has no impact on osteoblast functions [176–178] the observation that the addition of pegvisomant to pasireotide therapy prevented occurrence of VF suggests that disease control could be important for skeletal health besides the effect of individual medications [179, 180].

### Vitamin D balance

Active acromegaly is a risk factor for vitamin D deficiency correlating with IGF-I levels (LQ) [181]. Increased vitamin D binding protein likely reduces free vitamin D concentrations [182]. Low vitamin D concentrations might be associated with increased risk of i-VFs and supplementation with cholecalciferol may be protective from VF occurrence [183].

## Other comorbidities

### Consensus recommendations

*Diagnosis*:

- *Colonoscopy is recommended at diagnosis per country-specific general guidelines (SR)*
- *Screening for cancers*,* including thyroid*,* should follow country-specific general protocols (DR).*
- *Early joint clinical assessment is crucial to guide further radiological evaluation if needed (SR).*
- *Renal function (GFR) should be assessed at diagnosis (SR)*
- *QoL should be assessed with dedicated symptoms questionnaires (DR)*

*Therapy*:

- *In addition to normalizing biochemical parameters*,* improvement in QoL should be addressed*,* using a multidisciplinary*,* patient-centered approach which specifically addresses burden of treatment complications*,* and maladaptive coping (DR)*.
- *Physiotherapy and other patient-centered approaches to improve joint function and QoL should be integrated in the multidisciplinary management of arthropathy (DR)*.
- *Joint prostheses might be considered in specific patients (DR)*

*Follow up*:

- *Follow-up with colonoscopy is recommended as per country-specific general guidelines (SR)*.
- *Patient-reported outcome measures (PROMs*), *symptom burden and QoL and clinician-reported outcome tools (SAGIT instrument and Acromegaly Disease Activity Tool) have shown different relationships with biochemical outcomes and should be assessed during follow-up (DR)*.

### Cancer

A priori, GH and IGF-I play important roles in cancer development and progression (MQ) [184]. Genetic and/or epigenetic alterations in acromegaly, presence of comorbidities (insulin resistance and diabetes), and aging of this population due to increased survival rates also predispose to cancer risk (MQ) [185, 186]. However, there has been a long-standing debate as to whether acromegaly has a higher risk of malignancy compared to general population.

Improved overall survival rates have led to an increased incidence of reported age-related malignancies (LQ). The incidence of cancer, particularly colon and thyroid cancers has increased mainly according to different reports which might have selection biases [185, 187, 188]. Population-based studies with lesser bias also revealed inconsistent results, some with no increase in cancer incidence [189] as opposed to others with a slight increase [187, 190]. However, large-scale studies show increased cancer incidence [39, 191]. In contrast, no increased incidence was found in malignancy-associated mortality [39]. In a recent, prospective, longitudinal large cohort study, cancer incidence was increased and cumulative exposure to IGF-I excess was a cancer predictor [192]. The total number of cancer cases observed was 156 and the expected number of cases was 87, yielding an increased standardized incidence ratio (SIR) of 1.78 (95% CI, 1.51–1.81). The SIR was increased for thyroid, 6.87 (4.3-10.05); breast, 1.67 (1.16–2.26); and colorectal, 2.65 (1.41–4.29) cancers. Trends for increases in SIR were found for renal, 1.67 (0.59–3.27); and prostate, 1.5 (0.89–2.27) cancers. Population-based genetic, geographic, and other factors may explain the heterogeneity of the results [192].

### Quality of life

Despite advances in diagnostic modalities, a significant delay in acromegaly diagnosis persists, with patients often experiencing symptoms for several years prior to diagnosis (HQ). This latency contributes to irreversible acromegaly-associated cardiovascular disease, secondary diabetes mellitus, hypopituitarism, arthropathy, VFs, and psychological disorders [193, 194] (HQ). Impaired QoL and disease activity do not correlate. Dissociated biochemical outcomes and PROMs is present in approximately one-third of patients (MQ).

Many of the acromegaly complications are associated with increased mortality and substantial impairment in QoL [195]. The use of the *Acromegaly Quality of Life questionnaire* (AcroQoL) and simultaneous generic questionnaires with normative data has confirmed that QoL is impaired and largely independent of biochemical outcomes, although an improvement was seen after optimal treatment of acromegaly [196]. AcroQoL impairment correlated with adverse psychological well-being and advancing age. Musculoskeletal disorders, arthropathy, and muscle weakness are particularly linked to impaired QoL (MQ).

QoL improves after long-term biochemical control, regardless of the treatment modality (HQ). In addition, optimizing treatment of hormone deficiencies improves QoL (MQ). Surgical adenoma resection is associated with greater improvements in QoL. While pharmacological treatment ameliorates both acromegaly-related comorbidities and QoL, the chronic requirement for monthly SRL injections is an adverse subjective perception of well-being [197]. Most patients treated with injectable SRLs and classified as well-controlled persistently experience acromegaly-related symptoms (MQ). Furthermore, most patients reported that these symptoms interfered with daily functioning and adversely affected both leisure and occupational activities. Gastrointestinal side effects and injection site reactions were also frequently reported, highlighting the persistent treatment burden associated with long-term injectable therapy [198]. Addition of pegvisomant to long-acting SRL therapy improved GH-dependent parameters of QoL and reported headache, soft-tissue swelling, and the physical domain of AcroQoL, irrespective of IGF-I control [196, 197, 199–201]. Impaired QoL determinants include longer disease duration, GHD, conventional radiotherapy, pain (mainly due to arthropathy), anxiety, depressive symptoms, impairments in cognitive functioning, and individual characteristics including older age at onset, female gender, and higher BMI (MQ) [196, 201].

Perceived discordance in patient- and physician-reported symptom frequency and severity, and injection site reactions which may not be relevant particularly with oral therapies [202, 203], underscore the need for better communication to improve care (LQ) [204]. PROMs (symptom burden and QoL) and clinician-reported outcome tools (SAGIT instrument and Acromegaly Disease Activity Tool) [205] which evaluate treatment efficacy and support shared decision making, have shown different relationships with biochemical outcomes [200]. Novel PROMs or combination of preexisting generic and disease specific PROMs are necessary for a patient-centered approach to achieve optimal QoL outcomes [206]. Patient-centered care requires acknowledging patients’ perspectives and promoting shared decision-making. Anxiety and depression should be assessed and monitored [195, 207].

### Arthropathy

Joint pain, stiffness, and functional impairment are closely associated with reduced QoL (HQ). The prevalence of arthropathy is 4–12 fold higher in acromegaly and GH and IGF-I excess is associated with specific joint changes and arthropathy (HQ) [79, 208]. Therefore, early assessment of joints is recommended (SR). Arthropathy pathogenesis involves both GH and IGF-I excess which promotes chondrocyte DNA synthesis, cell replication, proteoglycan, and glycosaminoglycan synthesis [209]. Initially, elevated GH and IGF-I concentrations induce cartilage hypertrophy and ligamentous laxity, joint hypermobility, joint space widening, and periarticular soft tissue hypertrophy, that may be partially reversible with treatment. With prolonged GH excess, a degenerative phase ensues, with osteophyte and cyst formation, fibrocartilage overgrowth, and irreversible joint damage, often unresponsive to medical therapy [210]. The unique imaging phenotype of arthropathy in acromegaly differs from that of primary osteoarthritis (OA) and is characterized by osteophytosis with widened joint spaces reflecting cartilage hypertrophy [208, 210, 211]. It is predominantly characterized by thicker cartilage and changes in biochemical cartilage composition [210]. Persistent acromegaly shows joint space narrowing (JSN) with more severe joint complaints [212]. JSN affects 10–15% of patients with controlled acromegaly, especially women and older patients. JSN occurs in active disease, characterized by higher pretreatment IGF-I and longer exposure to hormonal excess [213]. Prompt diagnosis and earlier treatment may improve arthropathy reversibility (MQ). SRLs may reverse joint thickening in the early stages of disease [214]. Over time, however, joint degeneration may progress despite biochemical control, with radiological progression seen even after long-term disease control (LQ). These observations could be the consequence of smoldering or residual disease activity, or perhaps a direct effect of SRLs since SSTR are expressed in joint and cartilage cells [211].

Degenerative and inflammatory joint diseases should be distinguished at diagnosis. Arthropathy pain is a prominent symptom adversely affecting QoL and can result in significant deterioration of function over time [213, 215]. Acromegaly patients displayed significantly worse scores in all Western Ontario and McMaster universities osteoarthritis index (WOMAC) items, and these correlate strongly with other disability questionnaires, with QoL, percentage of worktime loss and perceived impact on work productivity and on regular daily activities [213]. The high prevalence of depression is associated with female sex, and particularly the presence of arthropathy, which independently impairs QoL [215].

Persistence and progression of arthropathy occur despite sustained long-term disease management (MQ). Arthropathy should be treated as in general population. However, clinical and radiological findings of acromegaly-associated arthropathy differ from those of primary OA [210], and should prompt patient-centered approaches such as occupational health service for job reclassification, supervised appropriate physical activity and consideration by disability services. For persistent, disabling end-stage arthropathy, joint replacement or other surgical interventions may be considered, if the patient is in remission or well controlled biochemically (DR).

### Kidney

Acromegaly is associated with renal hypertrophy, renal cysts, elevated glomerular filtration rate (GFR) due to hyperfiltration, and microalbuminuria (HQ). Acromegaly is characterized by changes in renal structure and function, including variable expression of GH receptor, IGF-I, IGF-IR, and IGF-I-binding proteins variably expressed in anatomically and functionally different kidney segments [216]. Exposure to chronic GH and IGF-I excess increases renal plasma flow, GFR, and renal size [216]. These alterations may revert only partially after correction of GH and IGF-I excess (MQ). There is an increased prevalence of renal cysts and kidney length compared to the age-sex matched healthy population [217]. A 4.35-fold higher risk of developing end-stage kidney disease (ESKD) has been shown [218], also mediated by associated diabetes and hypertension. These reports underscore the need for timely, comprehensive management and long-term follow-up to mitigate ESKD risk. Increased risk of renal impairment and renal/ureteric cancer is reported (LQ). Few studies reported increased risk of kidney cancer [186, 219].

GH exerts an anti-natriuretic effect leading to sodium and water retention with consequent increase in extracellular water (MQ). Mechanisms underlying antinatriuretic action of the GH/IGF-I axis include renal artery stenosis, changes in antinatriuretic peptides, and ENaC regulation (MQ). Sodium overload may be alleviated by coadministration of the ENaC blocker amiloride [24]. Increased body water and sodium, responsible for soft tissue swelling, leads to a broad spectrum of complications and may lead to high mortality in untreated patients (LQ).

Increased calcitriol and direct effect of IGF-I on the proximal tubule predispose to hyperphosphatemia (MQ). Hypercalciuria, mild hyperphosphatemia, and mild rise in plasma calcium are observed in active vs. treated acromegaly (MQ). Decreased fractional sodium and potassium excretion, hypercalciuria, hyperphosphaturia, microalbuminuria and high prevalence of micronephrolithiasis typically occur [220]. IGF-I-mediated and PTH-independent increased in calcitriol concentrations enhances intestinal calcium absorption and elevated fasting plasma calcium concentrations, the latter linked to increased distal tubular calcium reabsorption via the TRPV5 epithelial calcium channel [221]. Increased plasma phosphate concentrations are associated with both increased calcitriol-driven dietary phosphate absorption and direct antiphosphaturic action of IGF-I in the proximal tubule [24].

Effective treatment of acromegaly leads to rapid improvements in fluid retention, myocardial and peripheral nerve edema, improved cardiac function and reduction of misdiagnosed neuropathies (MQ). OSAS, partly due to soft tissue swelling, also improves with biochemical control (MQ). Since dysmorphic features may improve promptly after successful surgery or medical treatment, underlying the importance of water retention in the acromegaly phenotype (MQ) [24].

## Treatment impacts on acromegaly complications

### Surgery

If surgery is performed in dedicated neurosurgical centers by experienced neurosurgeons [14], beneficial effects are prompt (HQ). Surgery improves metabolic and cardiorespiratory disorders [88, 111, 222]. Postoperative remission is more common in older patients and those with preserved anterior pituitary function [113]. After surgery, improved glucose tolerance was observed in 87.3% of patients with impaired glucose tolerance and in 66.7% of those with diabetes mellitus. In contrast, deterioration in glucose tolerance occurred in 14.3% of patients with normal glucose tolerance. Improvement was more likely in individuals with lower preoperative FPG, 2-hour blood glucose, and HbA1c concentrations, as well as HOMA-β and insulinogenic index to insulin resistance ratio [112, 223]. Headache, visual acuity compromise, visual field impairment and cranial nerve palsies also ameliorate or resolve completely [224].

Successful transsphenoidal surgical resection improves ventricular mass and diastolic function, and modestly decreases BP and, in some cases, normalizes circadian BP rhythm [55]. Improved OSAS severity evaluated by polysomnography after surgery was associated with decreased IGF-I concentrations [88]. Surgery also ameliorates synovial thickening, bone marrow lesions [225], and peripheral nerve compression (carpal tunnel syndrome) [226, 227]. In experienced neurosurgical centers, regaining normal anterior pituitary function after surgery occurs more often than induction of new pituitary deficiency [228].

Pituitary adenectomy was more effective than SRLs in reducing mortality [229]. The concurrent presence of at least three cardiovascular risk factors was more frequently observed in patients receiving SRLs compared to those undergoing surgical remission [230]. QoL improvement is more pronounced with surgery compared to SRLs [212].

### Somatostatin receptor ligands

Information on long-term efficacy and safety is derived from studies investigating the use of long-acting octreotide and lanreotide preparations which improve LVM, LVEF, heart rate and arrhythmias (HQ) [222, 231]. Fg-SRLs have both positive and negative impacts on glucose metabolism, because in addition to inhibiting GH, they may also suppress glucagon, gastrointestinal glucose absorption, hepatic glucose production, and insulin secretion (MQ) [230]. While these agents have a generally neutral effect on glucose metabolism [114], post glucose insulin suppression occurs and may lead to progressive glucose tolerance impairment, independently of the degree of GH/IGF-I control. Improved insulin sensitivity and reduced insulin secretion may lead to hypoglycemic and/or hyperglycemic episodes, highlighting the importance of glucose monitoring [4, 230]. OSAS resolves or improves in patients achieving biochemical remission with fg-SRLs [85, 87], most significantly within the first year of treatment [89]. Although partial reversibility of cartilage hypertrophy after short-term fg-SRL therapy was shown, arthropathy usually persists despite long-term biochemical remission in most patients [212].

Pasireotide is slightly more effective in normalizing GH and IGF-I concentrations improving headache and reducing adenoma-size (MQ). Pasireotide reduces insulin and incretin secretion, with impaired glucose tolerance sometimes warranting diabetic medication initiation or dose change (MQ) [117]. In patients with active disease, incidental VFs occurred less frequently with pasireotide compared to pegvisomant, despite similar IGF-I concentrations [175].

### Pegvisomant

Pegvisomant reduces cardiovascular complications and OSAS prevalence (HQ). In two large registries, pegvisomant improved BP, reducing the risk for coronary heart disease [232], as well as increased LVEF and decreased LVM index (MQ) [67]. The prevalence of cardiac rhythm disturbances decreased from 15% to 7.7% after long-term pegvisomant [78]. Treatment with pegvisomant reduces tongue volume and the severity of OSAS, which resolves in around 50% of patients [67, 86]. Pegvisomant uniquely improves insulin sensitivity, independently of disease control and reduces the need for antidiabetic medications (MQ) [233]. Pegvisomant significantly decreased FPG, HbA1c, fasting plasma insulin, and HOMA-IR. Addition of pegvisomant to SRLs alleviates adverse SRL effects on glucose metabolism [122].

## Conclusion

Contemporary management of acromegaly and its comorbidities has yielded lower mortality rates, approaching those of the general population. Early diagnosis and prompt achievement of biochemical remission are *sine qua non* for improved QoL, especially at experienced centers with dedicated neurosurgeons at a PTCOE [14, 15]. These centers provide multimodal management with a personalized approach to medical treatment options, as well as access to specialists for diagnosis, monitoring, and treatment of disease related comorbidities [216, 217]. Specific and effective treatments for systemic comorbidities of the disease play a key clinical role in all patients with acromegaly [218].

## Data Availability

No datasets were generated or analysed during the current study.
